# Association between frailty and gestational diabetes mellitus: a bidirectional and multivariable Mendelian randomization study

**DOI:** 10.3389/fendo.2024.1382516

**Published:** 2024-06-27

**Authors:** Xiao Li, Rui Xiong

**Affiliations:** ^1^ Obstetrics and Gynecology, Mianyang Central Hospital, Mianyang, Sichuan, China; ^2^ Obstetrics and Gynecology, Chengdu Xinhua Hospital Affiliated To North Sichuan Medical College, Chengdu, Sichuan, China

**Keywords:** frailty, gestational diabetes mellitus, Mendelian randomization, UK Biobank, FinnGen

## Abstract

**Background:**

The causality between frailty and gestational diabetes mellitus (GDM) has not yet been fully explored. A potential bidirectional causality was also needed to be confirmed.

**Methods:**

A bidirectional two-sample Mendelian randomization (MR) was conducted, with frailty-related data was collected from UK Biobank and TwinGen and GDM-related data was collected from the FinnGen consortium. We performed univariable and multivariable-adjusted MR with adjustments for body mass index (BMI). Several methodologies of MR were conducted to confirm the robustness of results.

**Results:**

Frailty was significantly associated with elevated risks of GDM (OR, 3.563; 95% CI, 1.737 to 7.309; P< 0.001) and GDM was also significantly associated with elevated risks of frailty ( β , 0.087; 95% CI, 0.040 to 0.133; P< 0.001). There is no evidence demonstrating the existence of horizontal pleiotropy and heterogeneity. This association was robust after adjustments for BMI. The sensitivity analyses with Weighted median, Maximum likelihood, Penalised weighted median, MR Egger and MR PRESSO methods indicated consistent results.

**Conclusion:**

Our study provides evidence of the bidirectional causal association between frailty and GDM from genetic perspectives, signaling that the identification and assessment of frailty should become a standard strategy during the early stages and care of gestational diabetes.

## Introduction

Gestational diabetes mellitus (GDM) is one of the most common complications during pregnancy, affecting 14.2% of pregnancy individuals and having serious adverse effects on both maternal and infant health (1, 2). What’s more significant is the growing realization that GDM serves as a glimpse into future health, and not just an isolated of disease that concludes with delivery (3–7). For example, Women with a previous history of GDM are prone to cardiovascular disease and have a nearly 30% increased mortality risk (4, 7). Therefore, identifying modifiable factors that can be used to prevent disease at an early stage or to prevent serious complications is important in reducing the harm and burden of disease associated with GDM.

As an easily intervened factor, frailty is a state of vulnerability to poor resolution of homeostasis, which emerges as one of the most pressing global public health challenges we will face (8, 9). Individuals with heightened frailty levels are markedly more vulnerable to a range of adverse consequences, such as cardiovascular disease, neurological disorders, disability, and mortality, when compared to those with lower frailty levels (8, 10–13). Notably, pregnant women often undergo shifts in dietary habits, reduced physical activity, weight gain, and substantial fluctuations in hormone levels (14, 15). These changes can disrupt homeostasis in blood glucose and the overall internal environment, leading to a concurrent presence of frailty (14–17). However, no studies have estimated the relationship between GDM and frailty. Existing research have focused on the association between type 2 diabetes (T2D) and frailty. On the one hand, previous studies indicates that frailty symptoms contribute to the progression from prediabetes to T2D in adults (18, 19). On the other hand, abnormal blood glucose emerges as a pivotal risk factor for frailty development, with the prevalence of frailty syndrome surging from an average of 5 to 10 percent in nondiabetic patients to 32 to 48 percent in diabetic patients (20–22). The above epidemiologic findings imply a possible coexistence of GMD and frailty. The elusive causal relationship between frailty and GDM poses limitations to the effective management of these interconnected health challenges.

Although a randomized controlled trial (RCT) is widely considered the golden standard for establishing causality, it is not applicable to the current topic due to ethics (23). With the rapid advancements of genome-wide association studies (GWASs), Mendelian randomization (MR) is frequently employed to infer causality by utilizing phenotypic-associated single nucleotide polymorphisms (SNPs) as instrumental variables, which eliminating confounding bias and reverse causes and making the MR method a “natural RCT” (24–26). Therefore, in this study, we hypothesized that there may be a directional causal effect between frailty and GDM and performed a bidirectional MR analysis between frailty and GDM using summary-level data to detect the exact causality.

## Methods

### Study design and data sources

**Figure 1:**
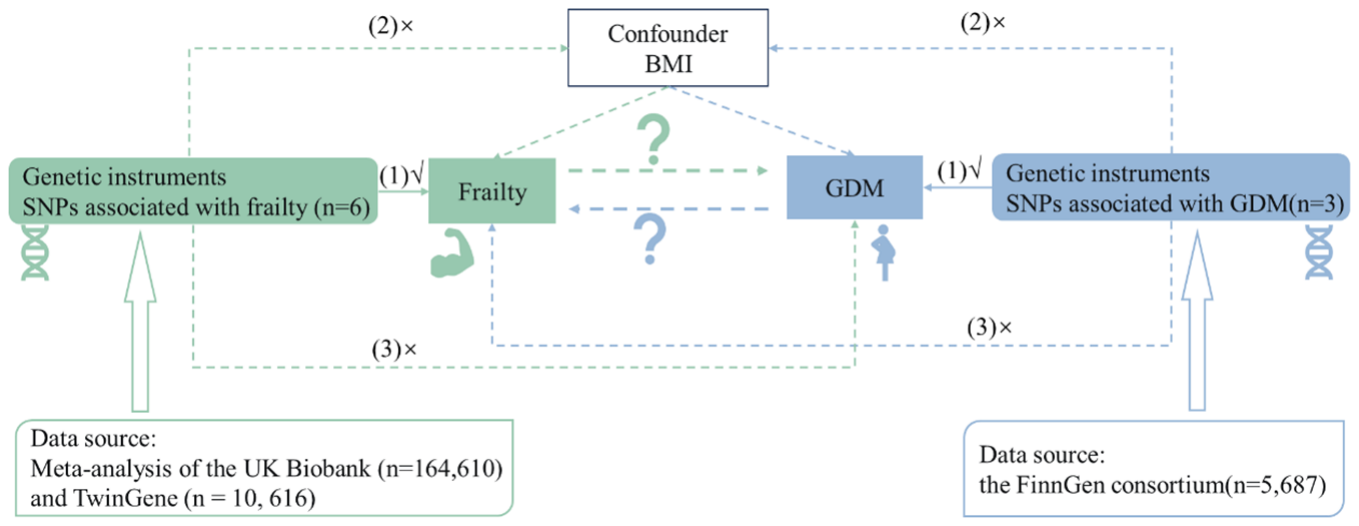
Flowchart of overall study design.

This research was engineered as a bidirectional two-sample MR study, with a comprehensive overview outlined in **Figure 1**. The frailty-related datasets used in the existing studies are publicly available, and ethical permission was granted for the original paper (27). An extensive GWAS meta-analysis provided SNPs associated with frailty. This incorporated European participants from the UK Biobank (n = 164, 610, aged between 60 and 70 years, 48.7% male) and TwinGene (n = 10, 616, aged between 41 and 87 years, 47.5% male). Frailty was measured by the frailty index, which is based on a collection of 49 health deficits over an individual’s lifetime (27, 28). This measurement tool is widely validated and frequently used in clinical settings (29). Information related to genetic variants associated with GDM was obtained from the FinnGen consortium as part of the ongoing Finnish national study initiated in 2017 (30, 31). The GDM dataset, with GWAS-ID of Finland-b-GEST_DIABETES, was obtained from the MRC-IEU. The dataset includes a total of 5,687 cases of GDM in 123,579 women, and the dataset consists exclusively of Europeans (30, 31).

### SNPs selection

In this study, we selected a robust threshold of P< 5 × 10^−8^ indicating genome-wide significant associations between SNPs and exposures. Subsequently, we employed the “clump_data” function to identify independent SNPs, utilizing a linkage disequilibrium (LD) cutoff value of R^2^ = 0.001 within a window of 10,000 kb (32). Moreover, the *F* statistic (33) was computed to ascertain the presence of a weak instrumental variable bias in the selected instrumental variables (IVs). A weak instrumental bias was screened if the *F* statistic no more than 10. In the end, after the harmonization of the exposure and outcome datasets, with the removal of palindromic and weak instrumental variants, the remaining SNPs were utilized for the execution of the MR analysis.

### Statistical analysis

The primary methodology for MR analysis was the inverse variance weighted (IVW) strategy. This strategy provides optimal effectiveness under the assumption of no average pleiotropic effect (34). To investigate potential heterogeneity resulting from varying genetic variants, we computed the Cochran’s *Q* statistic using IVW methods (34, 35). The presence of heterogeneity is indicated by a *P*-value< 0.05. If heterogeneity presents, we give effect estimates using IVW method under a multiplicative random effects framework. Furthermore, the intercept term of MR-Egger regression (36) was utilized to identify any horizontal pleiotropy, with deviation from zero (P value< 0.05) indicating directional pleiotropy.

To test the robustness of the results of the IVW method, we also employ several other well-established and horizontal pleiotropy robust methods, including MR-Egger (36), Penalised weighted median (37), weighted median (37), Maximum likelihood (38), and MR-PRESSO (39). We also conducted a Leave-One-Out (LOO) analysis (40) to determine if a specific single-nucleotide polymorphism (SNP) particularly influenced the aggregate effect. Furthermore, Multivariable Mendelian Randomization (MVMR) (41, 42), an expansion of MR that leverages genetic variants linked with multiple, potentially interconnected exposures, can identify the cumulative causal effects of numerous risk factors. In our research, BMI was adjusted during MVMR analyses because it was singled out as a significant confounding factor by PhenoScanner V2 (43).

## Results

### Characteristics of selected genetic variants

A total of 6 and 3 SNPs associated with frailty and GDM were selected according to the predetermined criteria, according to predefined criteria. The more detailed information of these SNPs is presented in **Supplementary Tables S1** and **S2**, respectively. The corresponding SNPs explained approximately 0.157% and 2.347% of total proportions of variance (R^2^) in frailty, and GDM, respectively. All *F* statistics exceeded 10, indicating a relatively low risk of weak instrument bias in the MR analyses conducted.

### Univariable MR analysis

#### Casual effect of frailty on gestational diabetes mellitus

**Figure 2:**
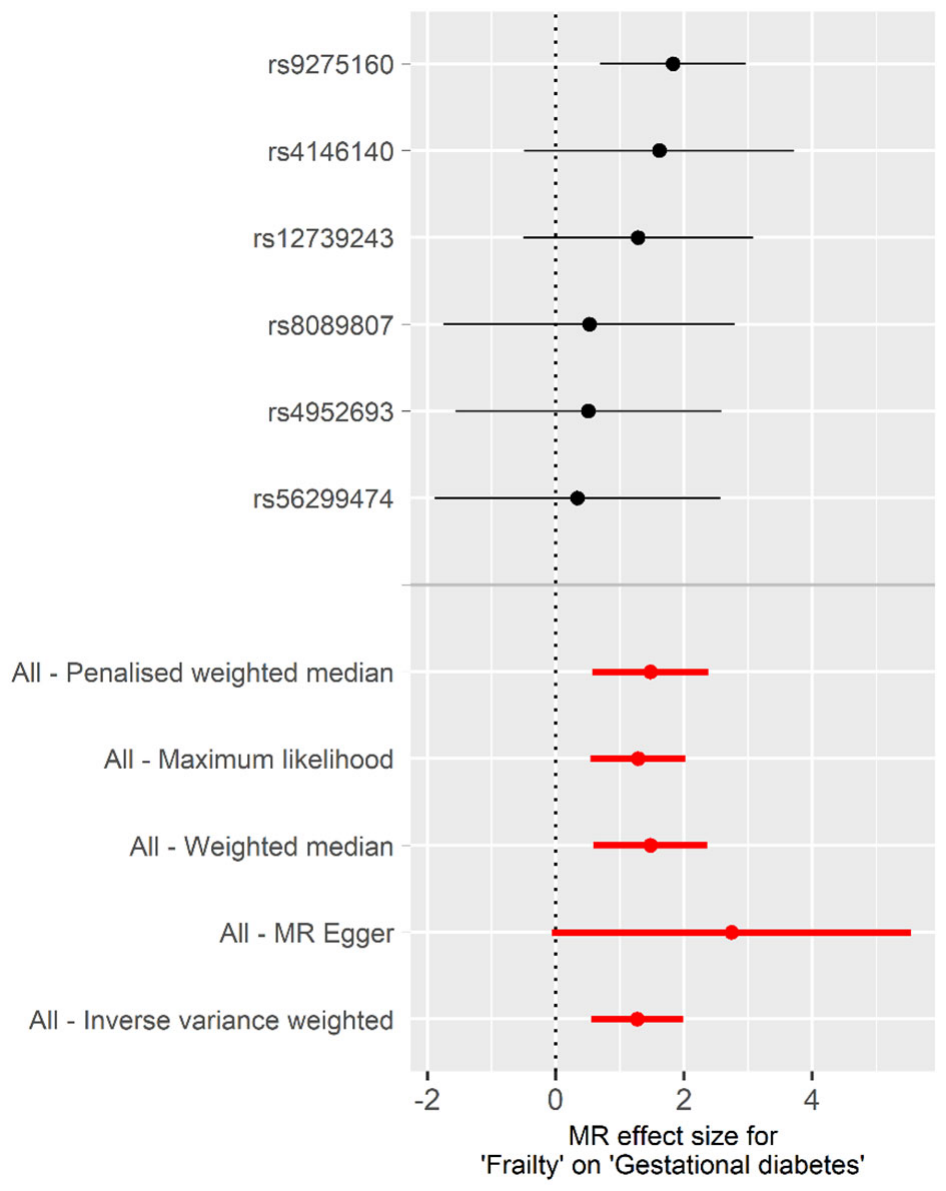
Forest plot of the individual and combined effect of frailty on GDM.

The univariable MR analysis to investigate the causal effect of frailty on GDM is shown in **Table 1**. Intercept term from the MR-Egger regression suggest no obvious directional pleiotropy among the SNPs in dataset, as the *P* values exceeded 0.05. No obvious heterogeneity was found in genetic variants associated with frailty and GDM (Cochran’s *Q* = 2.637 and *P* = 0.756). Thus, the IVW approach was employed under fixed effect to assess the causal effect of frailty with GDM. A higher frailty index was shown to correlate with an increased GBD risk [odds ratio (OR), 3.563; 95% CI, 1.737 to 7.309; *P*< 0.001]. This conclusion aligns with the outcomes from supplementary methods, including the weighted median, maximum likelihood, penalized weighted median, and MR-PRESSO, all demonstrated the risk effect of frailty on GBM (**Table 1**, **Figure 2**). These methods further substantiate the robustness of the results obtained via the IVW method.

**Table 1:** Univariate MR Estimates of Frailty on GDM.

Exposure	Outcome	Methods	*OR*	95% *CI*	*P*	*P* for heterogeneity	*P* for pleiotropy
Frailty	GDM	IVW	3.563	1.737–7.309	<0.001	0.756	
		Weighted median	4.368	1.738–10.976	0.002		
		Maximum likelihood	3.603	1.724–7.532	<0.001		
		Penalised weighted median	4.368	1.820–10.483	<0.001		
		MR Egger	15.512	0.994–255.019	0.127		0.347
		MR PRESSO	3.563	2.114–6.004	0.005		

**Supplementary Figure S1** displays scatter plots depicting the potential effects of SNPs on frailty in relation to GDM. The slope of each plot represents the evaluated effect size per method. Furthermore, the results of the LOO analysis are presented in **Supplementary Figure S2**, indicating that no single SNP is solely responsible for driving the overall effect. In addition, **Supplementary Figure S3** illustrates that the funnel plot was symmetrical.

#### Casual effect of gestational diabetes mellitus on frailty

**Figure 3:**
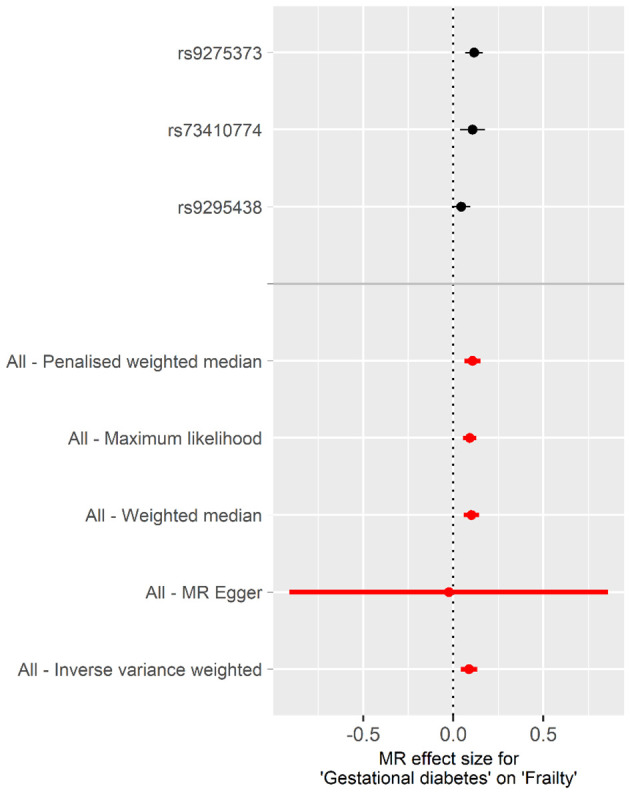
Forest plot of the individual and combined effect of GDM on frailty.

Using the genetic susceptibility to GDM as our exposures, the findings from reverse MR analyses are shown in **Table 2**. The absence of directional pleiotropy among the SNPs was indicated by the MR-Egger regression intercept term, with P values being higher than 0.05 (intercept = 0.025, P = 0.845). There was no obvious heterogeneity detected in genetic variants linked with GDM and frailty (Cochran’s Q = 4.283 and P = 0.117). As a result, the inverse variance weighted (IVW) method, under a fixed effect, was employed to examine any causal connections between GDM and frailty. The IVW method illustrated that pregnant woman with GDM have an increased frailty index [ β , 0.087; 95% confidence interval (CI), 0.040 to 0.133; P< 0.001]. When compared with the findings from our additional methods, including the weighted median, maximum likelihood, penalised weighted median, and MR-PRESSO, these all highlighted the potential risk effect of GDM on frailty, further substantiating the dependability of the results derived from the IVW method (**Table 2**, **Figure 3**).

**Table 2:** Univariate MR Estimates of GDM on Frailty.

Exposure	Outcome	Methods	β	95% *CI*	*P*	*P* for heterogeneity	*P* for pleiotropy
GDM	Frailty	IVW	0.087	0.040–0.133	<0.001	0.117	
		Weighted median	0.100	0.055–0.145	<0.001		
		Maximum likelihood	0.091	0.054–0.128	<0.001		
		Penalised weighted median	0.107	0.059 -0.154	<0.001		
		MR Egger	-0.025	-0.910–0.859	0.964		0.845
		MR PRESSO	0.075	0.022–0.128	<0.001		

Illustrated in **Supplementary Figure S4** are scatter plots that present the probable effects of SNPs on GDM, in connection with frailty. The inclination of each plot is indicative of the assessed effect size for every respective method. Outcomes from LOO analysis portrayed in **Supplementary Figure S5** suggest none of the single SNPs singlehandedly governs the total effect. Moreover, complementing this, **Supplementary Figure S6** demonstrates that the funnel plot was symmetrical.

### Multivariable MR analysis

Considering body mass index (BMI) was the major confounding factor in the association between frailty and GDM, we constructed multivariable MR (MVMR) adjusted for BMI to explore the bidirectional causal relationship between frailty and GDM, which presented in **Table 3**. The IVW method indicated that higher frailty index remained significantly associated with increased risks of GDM (OR, 2.183; 95% CI, 1.434 to 3.323; P< 0.001). Conversely, individuals with GDM were also more likely to have a higher frailty index (β, 0.025; 95% CI, 0.009 to 0.040; P = 0.002).

**Table 3:** MVMR Estimates between Frailty and GDM.

Exposure	Outcome	Methods	β/OR	95% *CI*	*P*
Frailty	GDM	IVW	2.183	1.434–3.323	<0.001
BMI	GDM	IVW	1.449	1.221–1.721	<0.001
GDM	Frailty	IVW	0.025	0.009–0.040	0.002
BMI	Frailty	IVW	0.235	0.205 -0.266	<0.001

## Discussion

To our understanding, this represents the premier systematic exploration concerning the correlation between frailty and GDM. In the bidirectional MR investigation undertaken, it was discerned that frailty manifested a positive causal impact on GDM prevalence. On the other hand, reverse direction analyses provided evidence that GDM was also positively associated with frailty. After adjusting for BMI for MVMR analysis, the above associations still robust. The implications of this study are meaningful, contributing extensively towards fortifying the health dynamics of both pregnant women and neonates.

Frailty is characterized by decreased functioning of multiple physiological systems, which increases the risk of adverse health outcomes and can occur at all ages (29, 44). Previous study on frailty and diabetes based on two prospective cohorts suggested that frailty was the predisposing factor for diabetes and increases its risk of death (11, 18). At present, there is a lack of studies on frailty and GDM. Considering the great influence of gestational diabetes on pregnant women and neonates, this study found that there is a causal relationship between frailty and gestational diabetes, which can provide information for the management of GDM. The above associations are likely to result from the loss of various biological reserves and the failure of homoeostatic mechanisms in the frailty state, and the detailed mechanisms remain to be explored (19).

On the other hand, it is worth noting that the bidirectional association between frailty and diabetes as well as GDM has not been solved (45, 46). The results of this study indicate that the population with GDM has a higher risk of frailty, which was consistent with existing mechanistic research findings. For example, it was found that women are like to undergo metabolic disorders before and during the course of their pregnancy, invisibly placing an increased amount of stress on beta cells (1). Additionally, GDM will also aggravate the insulin resistance in pregnancy, which is an important risk factor for frailty and would accelerate the progress in adverse events, endangering the long-term health of two generations (7).

Additionally, the association between frailty and gestational diabetes may be linked to the following endogenous factors: Firstly, cardiovascular diseases have been shown to be related to both frailty and gestational diabetes (3, 47–50); secondly, psychological factors such as depression may also mediate the relationship between the two (51, 52); lastly, malabsorption, celiac disease, and other nutrition-related problems may also be noteworthy factors to consider (53–55).

For the preservation of validity in the causal inference deriving from MR analyses, instrumental variables (SNPs) must adhere to three cornerstone assumptions. Firstly, under the “relevance assumption”, it is presupposed that a robust correlation exists between the genetic variants and the exposure phenotype. In striving to meet the assumptions, we confined our consideration to SNPs that had a significant correlation with exposure variables at a genome-wide level of significance (P< 5 × 10^-8^). Moreover, in order to ensure the strength of the instrument, we settled on SNPs with *F* statistics exceeding 10. Secondly, the “independence assumption” necessitates that instrumental variables are desirably devoid of any association with confounding. In addressing this, we have utilized PhenoScanner V2 (43) to eliminate certain SNPs potentially associated with confounding. Simultaneously, we have employed MVMR to control the confounding effects of BMI. Consequently, we observed that the results remain robust. Lastly, the “exclusion-restriction assumption” suggests that the route of causality should ideally traverse through the exposure of interest. In response to this, we implemented the MR-Egger approach, thereby confirming the absence of horizontal pleiotropy.

Our study possesses three distinct advantages. Firstly, it provides the premier comprehensive exploration of the reciprocal causality between frailty and gestational diabetes, affirming a bidirectional causal relationship between the two. Secondly, the utilization of PhenoScanner V2 and MVNR as two different analytical strategies effectively reduce the likelihood of potential confounding effects in MR studies. Lastly, we have employed various sensitivity analyses to verify the robustness of the IVW method results. Nevertheless, our study carries certain limitations. Firstly, our research scope is confined to the European populace due to the accessibility of GWAS data, thus extrapolation to other populations may encounter restrictions. Consequently, future investigations need to extend their research to diverse populations. Secondly, as we employed the GWAS summary data, we are incapable of stratification analysis based on demographic characteristics such as gender and age, which presents a possible direction for future research.

## Conclusion

This study confirms a bidirectional causal relationship between frailty and depression, signaling that the identification and assessment of frailty should become a standard strategy during the early stages and care of gestational diabetes.

## Data availability statement

The original contributions presented in the study are included in the article/**Supplementary Material**. Further inquiries can be directed to the corresponding author.

## Author contributions

XL: Writing – original draft. RX: Writing – review & editing.
