# Supplementary material for: Association between frailty and gestational diabetes mellitus: a bidirectional and multivariable Mendelian randomization study
Source: Front Endocrinol (Lausanne). 2024 Jun 27;15:1382516. doi: 10.3389/fendo.2024.1382516 (PMC11236542; doi:10.3389/fendo.2024.1382516)
Supplement: Supplementary file 1 [file DataSheet_1.docx]

Supplementary Materials for

**Association between frailty and gestational diabetes mellitus: A bidirectional Mendelian randomization study**

contents

[Supplementary figures 3](#_Toc157854181)

[Figure S1. Scatter plots of SNP effects on frailty versus GDM. 3](#_Toc157854182)

[Figure S2. The leave-one-out estimate of frailty on GDM. 4](#_Toc157854183)

[Figure S3. Funnel plot for frailty on GDM. 5](#_Toc157854184)

[Figure S4. Scatter plots of SNP effects on GDM versus frailty 6](#_Toc157854185)

[Figure S5. The leave-one-out estimate of GDM on frailty. 7](#_Toc157854186)

[Figure S6. Funnel plot for GDM on frailty. 8](#_Toc157854187)

[Supplementary tables 9](#_Toc157854188)

[Table S1. Characteristics of genetic variants used to estimate the effect of frailty on GDM. 9](#_Toc157854189)

[Table S2. Characteristics of genetic variants used to estimate the effect of GDM on frailty. 9](#_Toc157854190)

# Supplementary figures

## Figure S1. Scatter plots of SNP effects on frailty versus GDM.


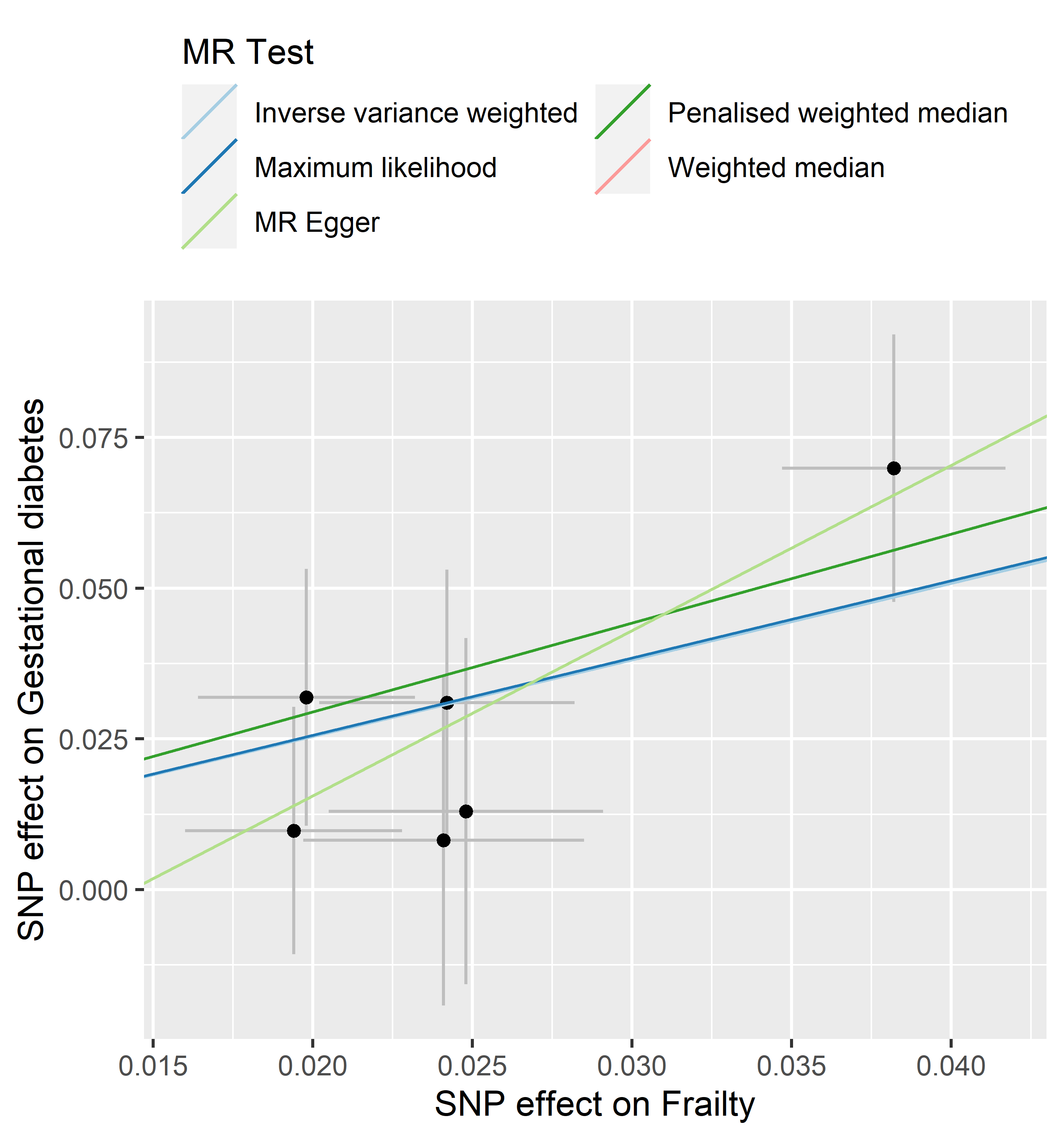


## Figure S2. The leave-one-out estimate of frailty on GDM.

Data are presented as β with the 95% confidence interval.


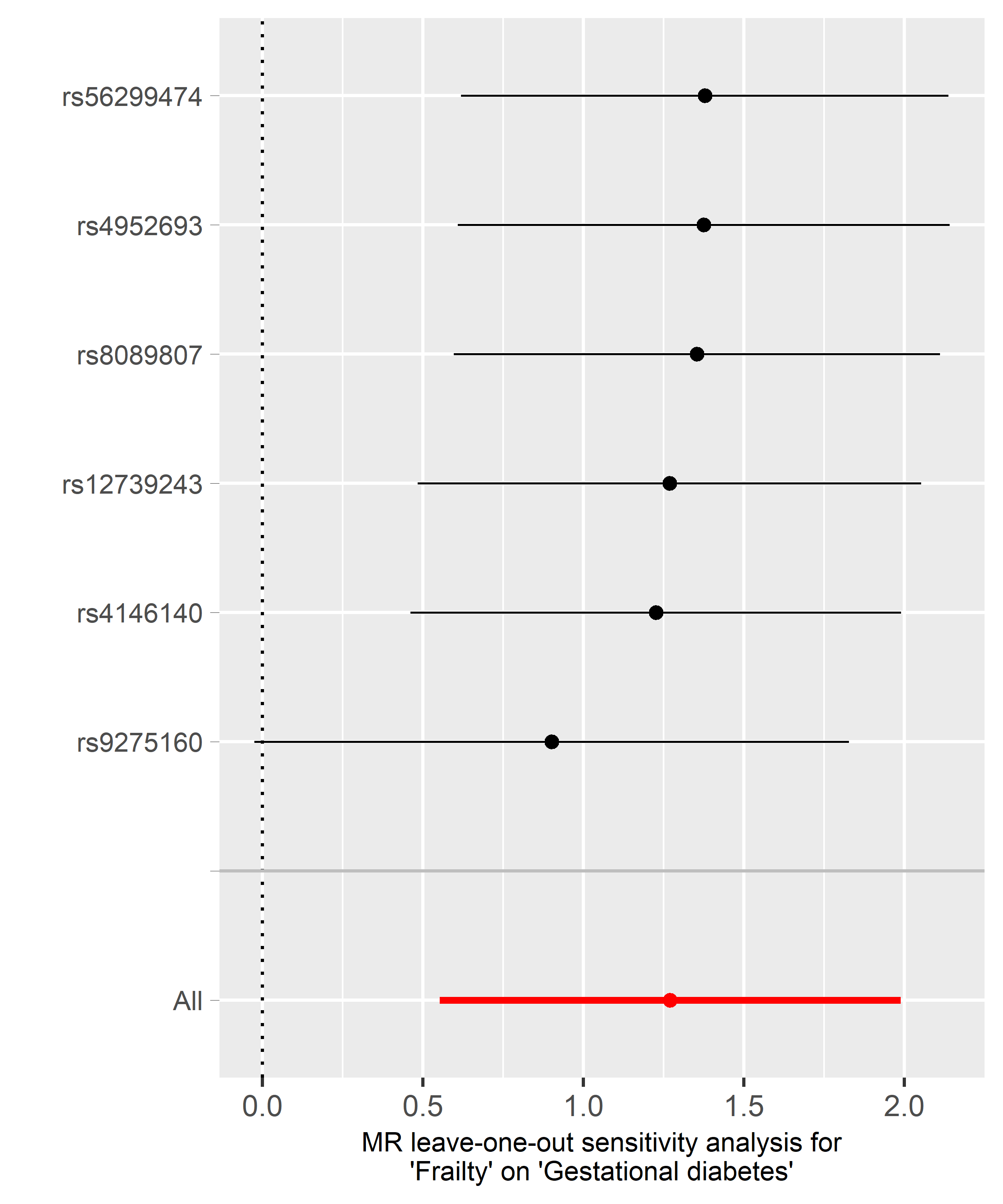


## Figure S3. Funnel plot for frailty on GDM.


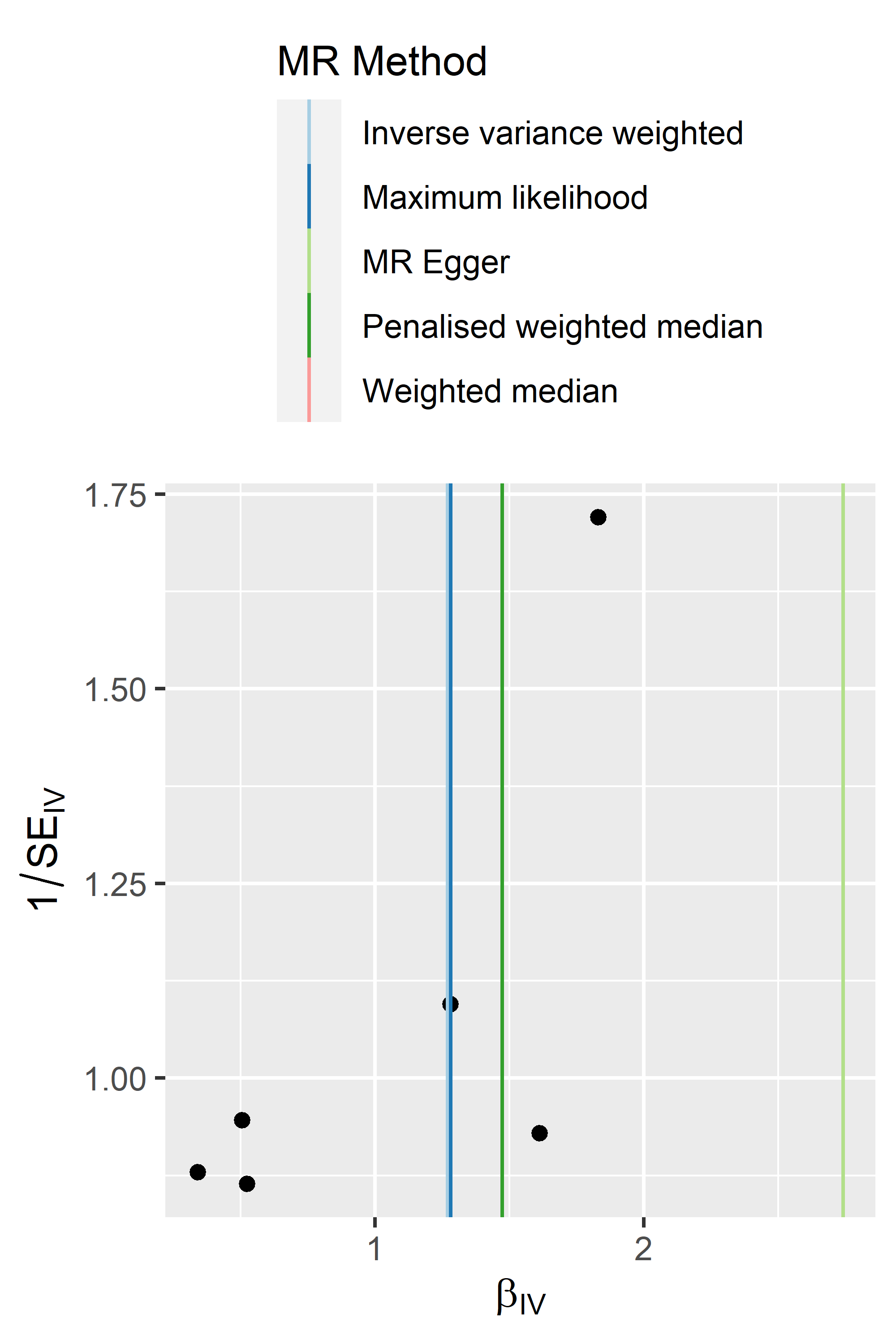


## Figure S4. Scatter plots of SNP effects on GDM versus frailty


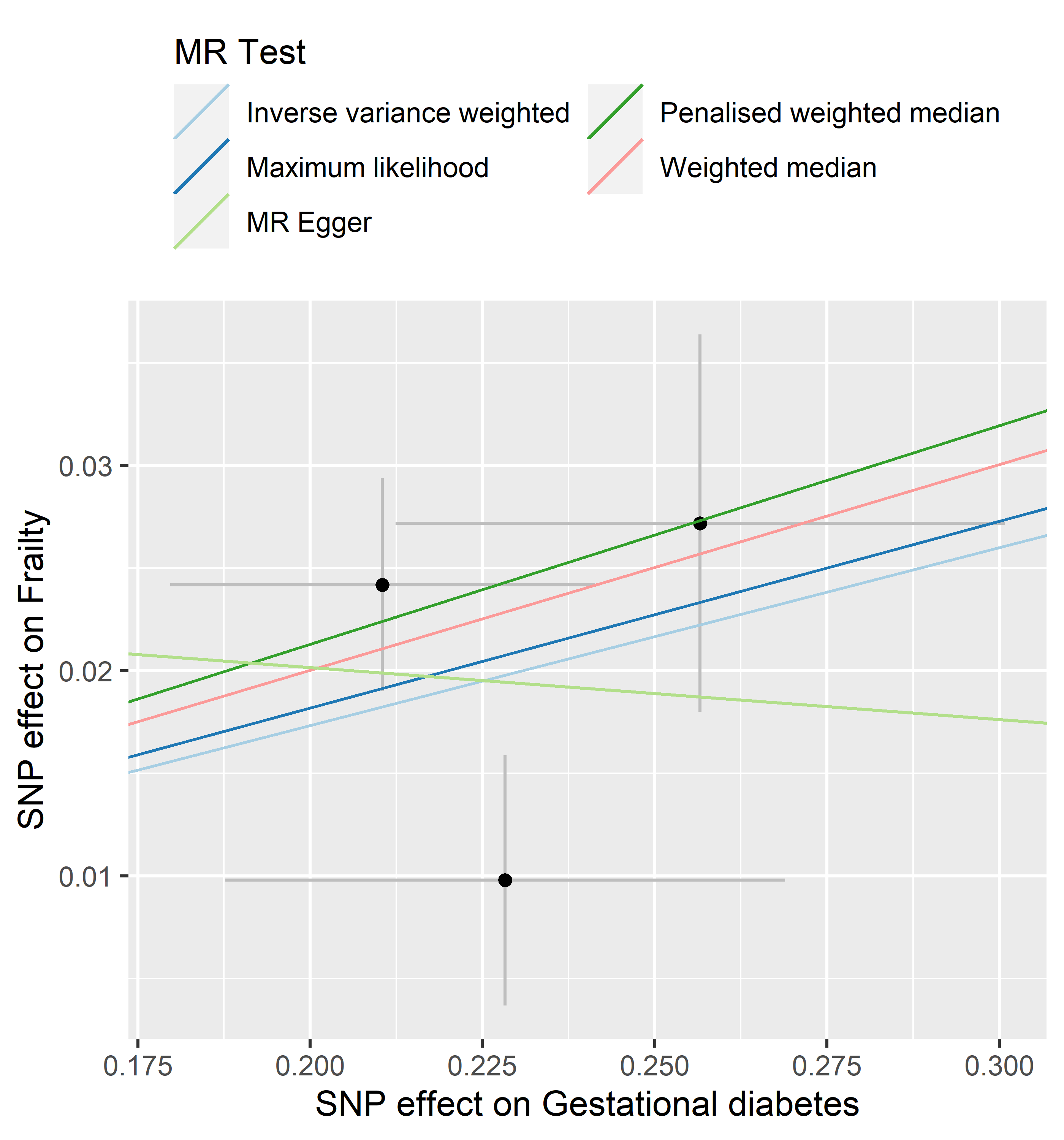


## Figure S5. The leave-one-out estimate of GDM on frailty.


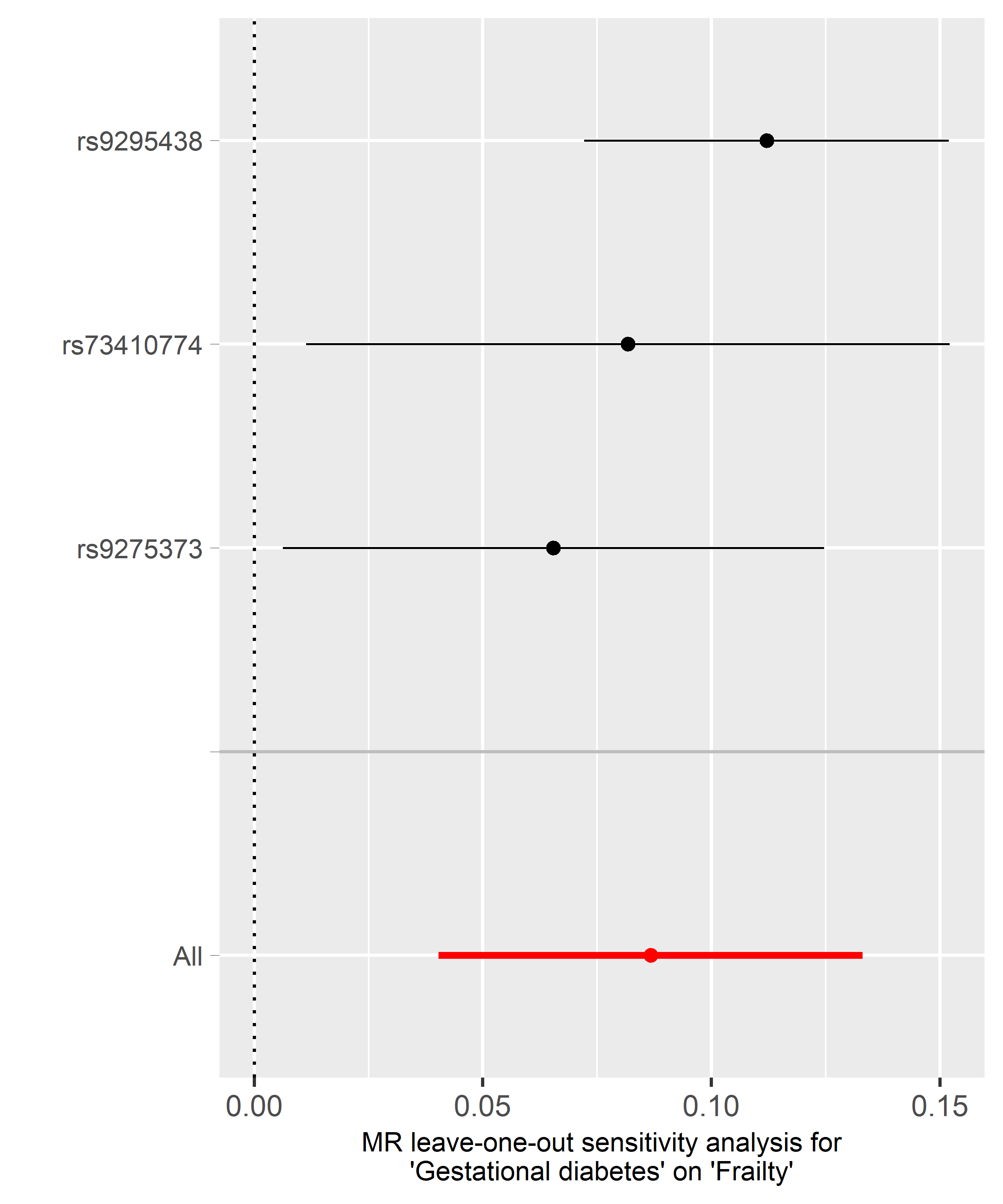


## Figure S6. Funnel plot for GDM on frailty.

Data are presented as β with the 95% confidence interval.


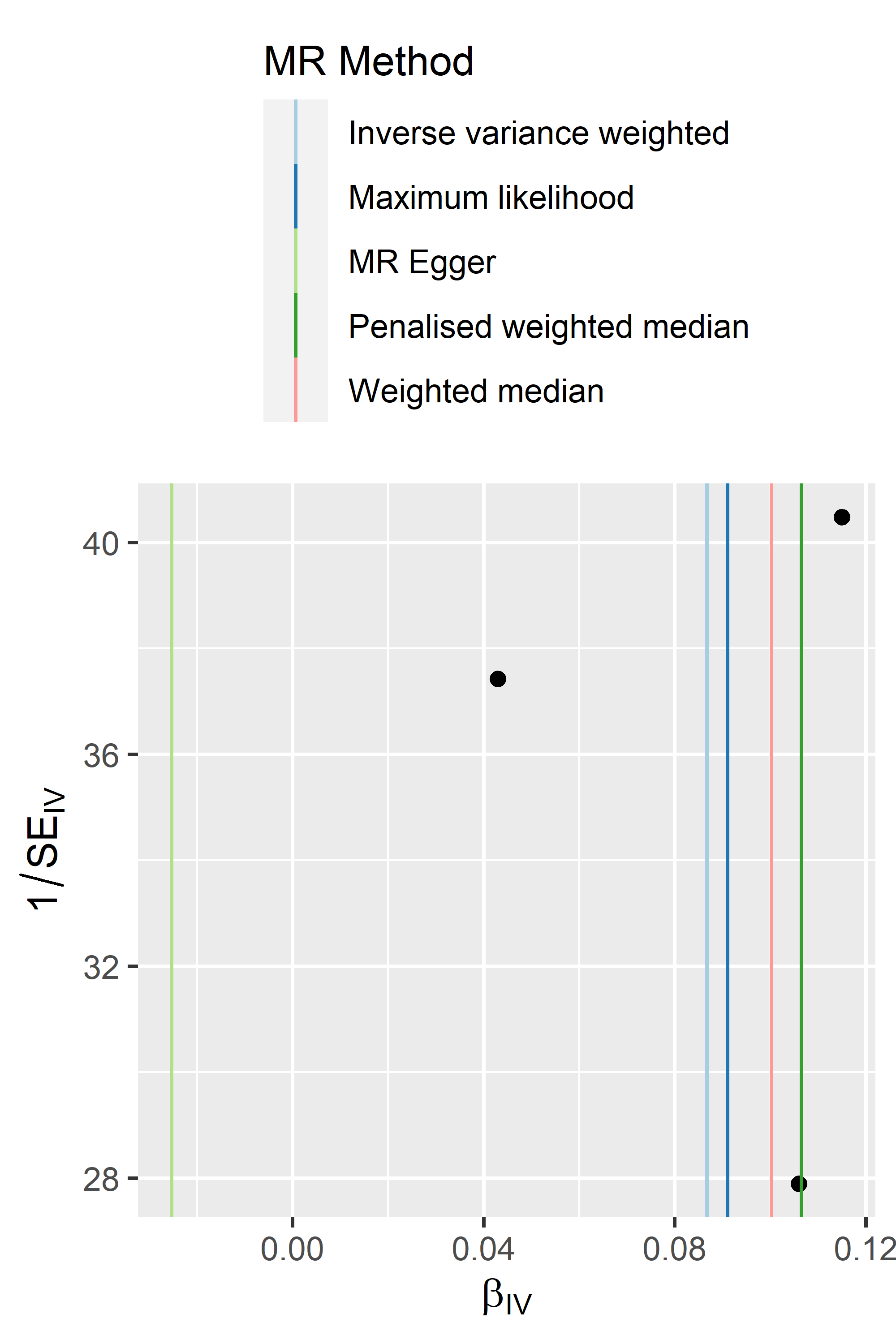


# Supplementary tables

## Table S1. Characteristics of genetic variants used to estimate the effect of frailty on GDM.

| **SNP** | **chr** | **pos** | **beta** | **se** | ***p*-val** | **A1** | **A2** | **eaf** | **R^2^** | **F** |
| --- | --- | --- | --- | --- | --- | --- | --- | --- | --- | --- |
| rs12739243 | 1 | 210302043 | -0.0242 | 0.004 | 1.28E-09 | C | T | 0.2206 | 0.000201385 | 35.29455385 |
| rs4952693 | 2 | 44151808 | -0.0194 | 0.0034 | 1.47E-08 | T | C | 0.3734 | 0.000176116 | 30.86513939 |
| rs9275160 | 6 | 32652620 | 0.0382 | 0.0035 | 7.18E-28 | A | G | 0.3397 | 0.000654626 | 114.7814084 |
| rs56299474 | 8 | 21992804 | 0.0241 | 0.0044 | 3.94E-08 | A | C | 0.1733 | 0.000166422 | 29.16597193 |
| rs4146140 | 10 | 61885362 | -0.0198 | 0.0034 | 6.83E-09 | T | C | 0.3811 | 0.000184935 | 32.41109633 |
| rs8089807 | 18 | 39322639 | -0.0248 | 0.0043 | 6.50E-09 | T | C | 0.1866 | 0.000186702 | 32.72079494 |

## Table S2. Characteristics of genetic variants used to estimate the effect of GDM on frailty.

| **SNP** | **chr** | **pos** | **beta** | **se** | ***p*-val** | **A1** | **A2** | **eaf** | **R^2^** | **F** |
| --- | --- | --- | --- | --- | --- | --- | --- | --- | --- | --- |
| rs9295438 | 6 | 19452664 | -0.2283 | 0.0406 | 1.85E-08 | C | T | 0.9245 | 0.00727605 | 905.7426827 |
| rs9275373 | 6 | 32668411 | 0.2105 | 0.0308 | 7.94E-12 | A | G | 0.1218 | 0.009479271 | 1182.630285 |
| rs73410774 | 6 | 32789739 | 0.2566 | 0.0442 | 6.29E-09 | T | G | 0.05388 | 0.006713007 | 835.1798532 |
