# Supplementary material for: Association between frailty and gestational diabetes mellitus: a bidirectional and multivariable Mendelian randomization study
Source: Front Endocrinol (Lausanne). 2024 Jun 27;15:1382516. doi: 10.3389/fendo.2024.1382516 (PMC11236542; doi:10.3389/fendo.2024.1382516)
Supplement: Supplementary file 2 [file DataSheet_2.docx]

**STROBE-MR checklist of recommended items to address in reports of Mendelian randomization studies**^1^ ^2^

| **Item No.** | **Section** | **Checklist item** | **Relevant text from manuscript** |
| --- | --- | --- | --- |
| 1 | **TITLE and ABSTRACT** | Indicate Mendelian randomization (MR) as the study’s design in the title and/or the abstract if that is a main purpose of the study | Association between frailty and gestational diabetes mellitus: a bidirectional and multivariable Mendelian randomization study |
|  | **INTRODUCTION** |  |  |
| 2 | **Background** | Explain the scientific background and rationale for the reported study. What is the exposure? Is a potential causal relationship between exposure and outcome plausible? Justify why MR is a helpful method to address the study question | Mendelian randomization (MR) is frequently employed to infer causality by utilizing phenotypic-associated single nucleotide polymorphisms (SNPs) as instrumental variables, which eliminating confounding bias and reverse causes and making the MR method a “natural RCT”. |
| 3 | **Objectives** | State specific objectives clearly, including pre-specified causal hypotheses (if any). State that MR is a method that, under specific assumptions, intends to estimate causal effects | Therefore, in this study, we hypothesized that there may be a directional causal effect between frailty and GDM and performed a bidirectional MR analysis between frailty and GDM using summary-level data to detect the exact causality. |
|  | **METHODS** |  |  |
| 4 | **Study design and data sources** | Present key elements of the study design early in the article. Consider including a table listing sources of data for all phases of the study. For each data source contributing to the analysis, describe the following: |  |
|  | a) | Setting: Describe the study design and the underlying population, if possible. Describe the setting, locations, and relevant dates, including periods of recruitment, exposure, follow-up, and data collection, when available. | The frailty-related datasets used in the existing studies are publicly available, and ethical permission was granted for the original paper…and the dataset consists exclusively of Europeans. |
|  | b) | Participants: Give the eligibility criteria, and the sources and methods of selection of participants. Report the sample size, and whether any power or sample size calculations were carried out prior to the main analysis | The frailty-related datasets used in the existing studies are publicly available, and ethical permission was granted for the original paper…and the dataset consists exclusively of Europeans. |
|  | c) | Describe measurement, quality control and selection of genetic variants | In this study, we selected a robust threshold of P < 5 × 10−8 indicating genome-wide significant associations between SNPs and exposures…the remaining SNPs were utilized for the execution of the MR analysis. |
|  | d) | For each exposure, outcome, and other relevant variables, describe methods of assessment and diagnostic criteria for diseases | The frailty-related datasets used in the existing studies are publicly available, and ethical permission was granted for the original paper…and the dataset consists exclusively of Europeans. |
|  | e) | Provide details of ethics committee approval and participant informed consent, if relevant | It is not relevant |
| 5 | **Assumptions** | Explicitly state the three core IV assumptions for the main analysis (relevance, independence and exclusion restriction) as well assumptions for any additional or sensitivity analysis | For the preservation of validity in the causal inference deriving from MR analyses, instrumental variables (SNPs) must adhere to three cornerstone assumptions…In response to this, we implemented the MR-Egger approach, thereby confirming the absence of horizontal pleiotropy. |
| 6 | **Statistical methods: main analysis** | Describe statistical methods and statistics used |  |
|  | a) | Describe how quantitative variables were handled in the analyses (i.e., scale, units, model) | The frailty-related datasets used in the existing studies are publicly available, and ethical permission was granted for the original paper…and the dataset consists exclusively of Europeans. |
|  | b) | Describe how genetic variants were handled in the analyses and, if applicable, how their weights were selected | The primary methodology for MR analysis was the inverse variance weighted (IVW) strategy…because it was singled out as a significant confounding factor by PhenoScanner V2. |
|  | c) | Describe the MR estimator (e.g. two-stage least squares, Wald ratio) and related statistics. Detail the included covariates and, in case of two-sample MR, whether the same covariate set was used for adjustment in the two samples | The primary methodology for MR analysis was the inverse variance weighted (IVW) strategy…because it was singled out as a significant confounding factor by PhenoScanner V2. |
|  | d) | Explain how missing data were addressed | It is not relevant |
|  | e) | If applicable, indicate how multiple testing was addressed | It is not relevant |
| 7 | **Assessment of assumptions** | Describe any methods or prior knowledge used to assess the assumptions or justify their validity | For the preservation of validity in the causal inference deriving from MR analyses, instrumental variables (SNPs) must adhere to three cornerstone assumptions…In response to this, we implemented the MR-Egger approach, thereby confirming the absence of horizontal pleiotropy. |
| 8 | **Sensitivity analyses and additional analyses** | Describe any sensitivity analyses or additional analyses performed (e.g. comparison of effect estimates from different approaches, independent replication, bias analytic techniques, validation of instruments, simulations) | The primary methodology for MR analysis was the inverse variance weighted (IVW) strategy…because it was singled out as a significant confounding factor by PhenoScanner V2. |
| 9 | **Software and pre-registration** |  |  |
|  | a) | Name statistical software and package(s), including version and settings used | The primary methodology for MR analysis was the inverse variance weighted (IVW) strategy…because it was singled out as a significant confounding factor by PhenoScanner V2. |
|  | b) | State whether the study protocol and details were pre-registered (as well as when and where) | It is not relevant |
|  | **RESULTS** |  |  |
| 10 | **Descriptive data** |  |  |
|  | a) | Report the numbers of individuals at each stage of included studies and reasons for exclusion. Consider use of a flow diagram | This research was engineered as a bidirectional two-sample MR study, with a comprehensive overview outlined in Fig. 1. |
|  | b) | Report summary statistics for phenotypic exposure(s), outcome(s), and other relevant variables (e.g. means, SDs, proportions) | It is not relevant for summary data MR analysis. |
|  | c) | If the data sources include meta-analyses of previous studies, provide the assessments of heterogeneity across these studies | It is not relevant |
|  | d) | For two-sample MR:  i.  Provide justification of the similarity of the genetic variant-exposure associations between the exposure and outcome samples  ii.  Provide information on the number of individuals who overlap between the exposure and outcome studies | The frailty-related datasets used in the existing studies are publicly available, and ethical permission was granted for the original paper…and the dataset consists exclusively of Europeans. The corresponding SNPs explained approximately 0.157% and 2.347% of total proportions of variance (R2) in frailty, and GDM, respectively. |
| 11 | **Main results** |  |  |
|  | a) | Report the associations between genetic variant and exposure, and between genetic variant and outcome, preferably on an interpretable scale | The univariable MR analysis to investigate the causal effect of frailty on GDM is shown in Table 1…Conversely, individuals with GDM were also more likely to have a higher frailty index (β, 0.025; 95% CI, 0.009 to 0.040; P = 0.002). |
|  | b) | Report MR estimates of the relationship between exposure and outcome, and the measures of uncertainty from the MR analysis, on an interpretable scale, such as odds ratio or relative risk per SD difference | The univariable MR analysis to investigate the causal effect of frailty on GDM is shown in Table 1…Conversely, individuals with GDM were also more likely to have a higher frailty index (β, 0.025; 95% CI, 0.009 to 0.040; P = 0.002). |
|  | c) | If relevant, consider translating estimates of relative risk into absolute risk for a meaningful time period | It is not relevant |
|  | d) | Consider plots to visualize results (e.g. forest plot, scatterplot of associations between genetic variants and outcome versus between genetic variants and exposure) | All demonstrated the risk effect of frailty on GBM (Table 1，Figure 2).  These all highlighted the potential risk effect of GDM on frailty, further substantiating the dependability of the results derived from the IVW method (Table 2, Figure 3) |
| 12 | **Assessment of assumptions** |  |  |
|  | a) | Report the assessment of the validity of the assumptions | For the preservation of validity in the causal inference deriving from MR analyses, instrumental variables (SNPs) must adhere to three cornerstone assumptions…In response to this, we implemented the MR-Egger approach, thereby confirming the absence of horizontal pleiotropy. |
|  | b) | Report any additional statistics (e.g., assessments of heterogeneity across genetic variants, such as *I^2^*, Q statistic or E-value) | Table1, table2 |
| 13 | **Sensitivity analyses and additional analyses** |  |  |
|  | a) | Report any sensitivity analyses to assess the robustness of the main results to violations of the assumptions | The univariable MR analysis to investigate the causal effect of frailty on GDM is shown in Table 1…Conversely, individuals with GDM were also more likely to have a higher frailty index (β, 0.025; 95% CI, 0.009 to 0.040; P = 0.002). |
|  | b) | Report results from other sensitivity analyses or additional analyses | The univariable MR analysis to investigate the causal effect of frailty on GDM is shown in Table 1…Conversely, individuals with GDM were also more likely to have a higher frailty index (β, 0.025; 95% CI, 0.009 to 0.040; P = 0.002). |
|  | c) | Report any assessment of direction of causal relationship (e.g., bidirectional MR) | The univariable MR analysis to investigate the causal effect of frailty on GDM is shown in Table 1…Conversely, individuals with GDM were also more likely to have a higher frailty index (β, 0.025; 95% CI, 0.009 to 0.040; P = 0.002). |
|  | d) | When relevant, report and compare with estimates from non-MR analyses | It is not relevant. |
|  | e) | Consider additional plots to visualize results (e.g., leave-one-out analyses) | All demonstrated the risk effect of frailty on GBM (Table 1，Figure 2).  These all highlighted the potential risk effect of GDM on frailty, further substantiating the dependability of the results derived from the IVW method (Table 2, Figure 3) |
|  | **DISCUSSION** |  |  |
| 14 | **Key results** | Summarize key results with reference to study objectives | To our understanding, this represents the premier systematic exploration concerning the correlation between frailty and GDM…The implications of this study are meaningful, contributing extensively towards fortifying the health dynamics of both pregnant women and neonates. |
| 15 | **Limitations** | Discuss limitations of the study, taking into account the validity of the IV assumptions, other sources of potential bias, and imprecision. Discuss both direction and magnitude of any potential bias and any efforts to address them | Nevertheless, our study carries certain limitations…which presents a possible direction for future research. |
| 16 | **Interpretation** |  |  |
|  | a) | Meaning: Give a cautious overall interpretation of results in the context of their limitations and in comparison with other studies | Frailty is characterized by decreased functioning of multiple physiological systems…thereby confirming the absence of horizontal pleiotropy. |
|  | b) | Mechanism: Discuss underlying biological mechanisms that could drive a potential causal relationship between the investigated exposure and the outcome, and whether the gene-environment equivalence assumption is reasonable. Use causal language carefully, clarifying that IV estimates may provide causal effects only under certain assumptions | Frailty is characterized by decreased functioning of multiple physiological systems…thereby confirming the absence of horizontal pleiotropy. |
|  | c) | Clinical relevance: Discuss whether the results have clinical or public policy relevance, and to what extent they inform effect sizes of possible interventions | To our understanding, this represents the premier systematic exploration concerning the correlation between frailty and GDM…The implications of this study are meaningful, contributing extensively towards fortifying the health dynamics of both pregnant women and neonates.  This study confirms a bidirectional causal relationship between frailty and depression, signaling that the identification and assessment of frailty should become a standard strategy during the early stages and care of gestational diabetes. |
| 17 | **Generalizability** | Discuss the generalizability of the study results (a) to other populations, (b) across other exposure periods/timings, and (c) across other levels of exposure | Nevertheless, our study carries certain limitations…which presents a possible direction for future research. |
|  | **OTHER INFORMATION** |  |  |
| 18 | **Funding** | Describe sources of funding and the role of funders in the present study and, if applicable, sources of funding for the databases and original study or studies on which the present study is based | Funding  None |
| 19 | **Data and data sharing** | Provide the data used to perform all analyses or report where and how the data can be accessed, and reference these sources in the article. Provide the statistical code needed to reproduce the results in the article, or report whether the code is publicly accessible and if so, where | The original contributions presented in the study are included in the article. Further inquiries can be directed to the corresponding authors. |
| 20 | **Conflicts of Interest** | All authors should declare all potential conflicts of interest | The authors declare that the research was conducted in the absence of any commercial or financial relationships that could be construed as a potential conflict of interest. |

This checklist is copyrighted by the Equator Network under the Creative Commons Attribution 3.0 Unported (CC BY 3.0) license.

1. Skrivankova VW, Richmond RC, Woolf BAR, Yarmolinsky J, Davies NM, Swanson SA, et al. Strengthening the Reporting of Observational Studies in Epidemiology using Mendelian Randomization (STROBE-MR) Statement. JAMA. 2021;under review.

2. Skrivankova VW, Richmond RC, Woolf BAR, Davies NM, Swanson SA, VanderWeele TJ, et al. Strengthening the Reporting of Observational Studies in Epidemiology using Mendelian Randomisation (STROBE-MR): Explanation and Elaboration. BMJ. 2021;375:n2233.
